# TLR7 agonism accelerates disease in a mouse model of primary Sjögren’s syndrome and drives expansion of T-bet^+^ B cells

**DOI:** 10.3389/fimmu.2022.1034336

**Published:** 2022-12-15

**Authors:** Achamaporn Punnanitinont, Eileen M. Kasperek, Jeremy Kiripolsky, Chengsong Zhu, Jeffrey C. Miecznikowski, Jill M. Kramer

**Affiliations:** ^1^ Department of Oral Biology, School of Dental Medicine, The University at Buffalo, State University of New York, Buffalo, NY, United States; ^2^ Department of Immunology, Microarray & Immune Phenotyping Core Facility, University of Texas Southwestern Medical Center, Dallas, TX, United States; ^3^ Department of Biostatistics, School of Public Health and Health Professions, The University at Buffalo, State University of New York, Buffalo, NY, United States

**Keywords:** autoantibodies, NOD.B10, autoimmunity, age-associated B cells, ABC, sialadenitis

## Abstract

Primary Sjögren’s syndrome (pSS) is a systemic autoimmune disease characterized by chronic inflammation of exocrine tissue, resulting in loss of tears and saliva. Patients also experience many extra-glandular disease manifestations. Treatment for pSS is palliative, and there are currently no treatments available that target disease etiology. Previous studies in our lab demonstrated that MyD88 is crucial for pSS pathogenesis in the NOD.B10Sn-*H2^b^* (NOD.B10) pSS mouse model, although the way in which MyD88-dependent pathways become activated in disease remains unknown. Based on its importance in other autoimmune diseases, we hypothesized that TLR7 activation accelerates pSS pathogenesis. We administered the TLR7 agonist Imiquimod (Imq) or sham treatment to pre-disease NOD.B10 females for 6 weeks. Parallel experiments were performed in age and sex-matched C57BL/10 controls. Imq-treated pSS animals exhibited cervical lymphadenopathy, splenomegaly, and expansion of TLR7-expressing B cells. Robust lymphocytic infiltration of exocrine tissues, kidney and lung was observed in pSS mice following treatment with Imq. TLR7 agonism also induced salivary hypofunction in pSS mice, which is a hallmark of disease. Anti-nuclear autoantibodies, including Ro (SSA) and La (SSB) were increased in pSS mice following Imq administration. Cervical lymph nodes from Imq-treated NOD.B10 animals demonstrated an increase in the percentage of activated/memory CD4+ T cells. Finally, T-bet^+^ B cells were expanded in the spleens of Imq-treated pSS mice. Thus, activation of TLR7 accelerates local and systemic disease and promotes expansion of T-bet-expressing B cells in pSS.

## 1 Introduction

Primary Sjögren’s syndrome (pSS, also referred to as Sjögren’s disease) is an autoimmune disease that primarily affects women. Patients with pSS experience many debilitating disease manifestations, including salivary hypofunction, diminished tear production, interstitial lung disease and nephritis (1). In addition, several hematopoietic abnormalities are noted, such as hypergammaglobulinemia and hypocomplementemia (2, 3) and pSS patients are at increased risk of B cell lymphoma development (4). Primary SS is incurable at present, although clinical trials are ongoing to discover effective therapeutics (5). Previous studies from our group and others highlight the importance of MyD88-mediated signaling cascades in specific disease manifestations (6–8), although the receptor-ligand interactions that culminate in MyD88-dependent disease sequelae remain incompletely understood. Indeed, additional studies are needed to define the molecular networks that contribute to disease initiation and persistence.

Both TLR and IL-1R family members drive MyD88-dependent inflammation, and TLR7 is a MyD88-dependent endosomal TLR that has been shown to be of critical importance in several autoimmune diseases (9–11), most notably Systemic Lupus Erythematosus (SLE) or lupus (12). Since SLE shares overlapping clinical and molecular features with pSS (13–16), we hypothesized that activation of TLR7 accelerates disease in a pSS mouse model.

Although TLR7 is expressed by diverse cells types, there is considerable evidence that the activation of TLR7-expressing B cells, in particular, is a central disease mechanism that drives SLE in mice and humans (17–20). TLR7 plays an integral role in host defense, as it elicits a protective response upon recognition of single-stranded viral RNA (21). Seminal studies revealed that self-derived ligands also activate TLR7, including RNA-associated immune complexes and U11 small nuclear RNA (U11snRNA), and this ability to recognize self-derived moieties underlies its pivotal role in autoimmunity (22–24).

Although there is compelling evidence that TLR7 is integral to lupus pathogenesis, there is a relative paucity of studies examining this TLR in pSS. Indeed, TLR7 activation is implicated in pSS patients, but little is known regarding its role in disease. Studies in pSS patients reveal TLR7 is expressed and elevated in salivary gland epithelial cells, minor salivary glands, and in parotid tissues from pSS patients (25–28). Additionally, TLR7 is dysregulated in the immune compartment, as levels are increased in PBMCs, B cells, and CD14^+^ monocytes from pSS patients (26, 28–31). Treatment of pSS B cells with a TLR7 agonist (CL264) caused elevated IFNα secretion as compared to B cells derived from healthy controls (30). Moreover, stimulation of TLR7 in pSS patient-derived naïve B cells using Imiquimod (Imq) resulted in increased plasma cell differentiation and class switching compared to B cells from healthy controls (32). Lastly, alterations in TLR7 signaling were identified in PBMCs from pSS patients using phosphorylation profiling (33).

Data from mouse models also provide corroborating evidence that TLR7 agonism mediates organ-specific disease (11, 34, 35). Studies in *TLR8-/-* animals revealed that these mice develop lupus and SS concomitantly, as sialadenitis, autoantibody production, immune complex deposition, salivary cytokine production, glomerulonephritis, and lung inflammation were observed (34, 36). These disease manifestations were dependent on TLR7, as disease was abrogated in mice lacking both TLR7 and TLR8 (34, 36). While these studies provide compelling evidence that TLR7 mediates SS, it is important to note that SS and lupus share overlapping disease features and so it is difficult to examine disease characteristics that result from SS specifically in the background of another autoimmune disease (15, 16). Further work in the NOD/ShiLtJ model revealed that TLR7 is required for lacrimal gland inflammation that is characteristic of SS (11). It is also challenging to assess SS-specific disease manifestations in the NOD/ShiLtJ mouse model, however, as these animals develop SS and type 1 diabetes (T1D), and hyperglycemia often accompanies T1D in this strain. Recent work revealed that hyperglycemia seen in the context of T1D influences salivary disease manifestations previously thought to be specific for SS (37, 38). Thus, given the potential of other autoimmune conditions to influence or confound interpretation of SS-related disease manifestations, studies are needed in pSS models to examine how TLR7 activation mediates SS-specific pathology.

To this end, we treated pre-disease stage (6-week-old) NOD.B10Sn-*H2^b^* (NOD.B10) pSS females with the TLR7 agonist Imiquimod (Imq) for 6 weeks, euthanized the animals, and evaluated local and systemic disease. Of note, we used a well-characterized pSS mouse model that develops clinical stage disease at 26 weeks of age. The use of pre-disease mice allowed us to determine if TLR7 activation accelerates disease, as animals normally display negligible signs of disease at 12 weeks of age (39). We performed parallel studies in age and sex-matched C57BL/10 (BL/10) healthy controls to assess whether the changes induced by Imq treatment were more robust in the pSS model. Strikingly, NOD.B10 mice exhibited cervical lymphadenopathy and splenomegaly following topical Imq treatment. Additionally, the percentage of TLR7-expressing cervical lymph node (cLN) B cells was expanded in NOD.B10 mice that received the TLR7 agonist. TLR7 agonism promoted exocrine-gland inflammation, and pSS mice treated with Imq exhibited a significant loss of salivary flow that was more pronounced than that observed in the sham treatment group. Pulmonary and renal inflammation were enhanced in NOD.B10 mice that received Imq, and total IgG was elevated in the sera of these animals compared to BL/10 females that also received Imq treatment. Autoantibodies were also enriched in pSS mice following TLR7 agonism. Finally, the percentage of activated/memory cLN CD4^+^ T cells were increased and T-bet^+^ B cells (also referred to as age-associated B cells or ABCs) were dramatically expanded in Imq-treated pSS animals. Thus, TLR7 agonism drives local and systemic pSS disease, and resulted in accelerated disease in the context of pSS.

## 2 Materials and methods

### 2.1 Mice

C57BL/10 (BL/10) (stock #000666) and NOD.B10Sn-*H2^b^* (NOD.B10) (stock #002591) were obtained from Jackson Labs. Animals were bred and maintained at the University of Buffalo laboratory animal facility in accordance with IACUC and NIH guidelines. All animals used in this study were female.

### 2.2 Treatment regimen

NOD.B10 female mice at a pre-disease stage (6 weeks of age) were given a sham base cream or 5% Imq cream. The Imq-containing cream was identical to the sham, with the exception of the addition of Imq. The treatments were administered epicutaneously to the ear three times a week for 6 weeks, as previously described (40). Parallel studies were performed using age and sex-matched healthy BL/10 controls. A detailed description of the treatment groups is provided in **Supplementary Table 1**.

### 2.3 Saliva and sera collection

Saliva was collected as previously described (7). Briefly, mice were injected with 0.3 mg/mL pilocarpine HCL (Sigma Aldrich) and saliva was collected for ten minutes on ice. Saliva was centrifuged and the volume assessed using a pipette. Saliva was collected prior to any treatment (5 weeks of age) and before euthanasia (12 weeks of age). Sera were harvested upon euthanasia by cardiac puncture. Blood was incubated at room temperature for 2 hours, and centrifuged at 0.7 g for 20 minutes. Sera were collected and frozen at -20 °C until use.

### 2.4 ELISAs

ELISAs were performed to quantify levels of IgM and IgG (Bethyl Laboratories), BAFF (R&D Systems), and β2-microglobulin (Lifespan Biosciences). Serial dilutions were prepared and values were calculated in accordance with manufacturer instructions. All samples were evaluated in duplicate.

### 2.5 HEp2 staining and autoantigen arrays

HEp2 staining was performed as previously described (7). Briefly, sera were diluted and incubated with HEp2 slides (Bion Enterprises) and goat anti-mouse IgG-Alexa 488 (Southern Biotech, 1.1 μg/mL) was used to detect the presence of anti-nuclear autoantibodies (ANAs). Slides were imaged using an Andor Dragonfly Microscope with a 20X glycerol objective (NA 0.75). Indirect immunofluorescence staining patterns were classified according to the ICAP using ImageJ after normalizing to the median intensity of each group (41). Autoantigen arrays were performed in collaboration with the UT Southwestern Genomics and Microarray Core Facility as previously described (7).

### 2.6 Flow cytometry

Spleens and cLNs were harvested and dissociated by mechanical dispersion. Following RBC lysis with ACK Lysis Buffer (Lonza), cells were incubated with Fc block (CD16/32, clone 2.4G2, BD Biosciences) and treated with antibodies directed against the following as indicated: B220 (clone RA3-6B2, BD Biosciences), CD23 (clone B3B4, Biolegend), CD21/35 (clone 7G6, BD Biosciences), T-bet (clone 4B10, BD Biosciences), CD11c (clone HL3, BD Biosciences), CD4 (clone GK1.5, BD Biosciences), CD8α (clone 53-6.7, BD Biosciences), CD44 (clone IM7, BD Biosciences), CD62L (clone MEL-14, BD Biosciences), CD138 (clone 281-2, BD Biosciences), CD69 (clone H1.2F3, Biolegend), and TLR7 (clone A94B10, BD Biosciences). Data were acquired using a BD Biosciences Fortessa and analyzed with FlowJo software (BD Biosciences).

### 2.7 Tissue collection and histological assessment

Submandibular salivary glands (SMGs), parotid salivary glands, lacrimal glands, lung and kidneys were harvested, tissue was fixed in 10% formalin and paraffin-embedded. Tissues were sectioned and stained with H&E. Slides were scanned using an Aperio ScanScope system (Leica Biosystems) with a 20X objective and 0.75 numerical aperture. Image/Fiji (version 1.53c) was used to measure the lymphocytic infiltration present in the tissues (42–44). The percent of lymphocytic infiltration was quantified by dividing the area of tissue occupied by lymphocytes by the total area of tissue examined and multiplying the value by 100.

### 2.8 Statistics

Autoantigen array data were analyzed using previously described methods (7). Briefly, for each comparison of two groups (BL/10 sham-treated versus BL/10 Imq-treated, NOD.B10 sham-treated versus NOD.B10 Imq-treated, BL/10 Imq-treated versus NOD.B10 Imq-treated, and BL/10 sham-treated versus NOD.B10 sham-treated) we first performed the two-sample t-test for all autoantigens, and then used the *p. adjust* R function in the stats R package to adjust the p-values in order to control the false discovery rate (45). The method proposed by Benjamini and Yekutieli was used in the adjustments (46). An autoantigen was deemed significant if the corresponding adjusted p-value was less than 0.05. The autoantigen array data is deposited in the Gene Expression Omnibus (GEO) database under the accession number GSE212467. Where appropriate, all other data were analyzed using paired student-T tests or ANOVA (Kruskal-Wallis test with multiple comparisons). All analyses were performed using the R programming language and Prism software (GraphPad) (47).

## 3 Results

### 3.1 Imq-treated pSS mice developed splenomegaly, cervical lymphadenopathy and expansion of TLR7-expressing B cells

**Figure 1:**
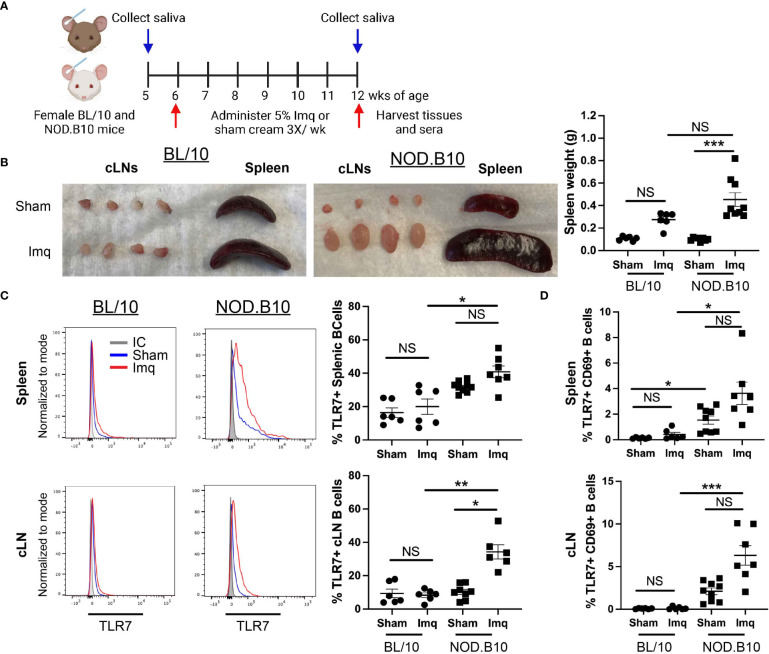
TLR7 agonism induces splenomegaly, cervical lymphadenopathy and expansion of TLR7-expressing B cells in NOD.B10 mice. **(A)** NOD.B10 and BL/10 mice were treated with sham or Imq-containing cream beginning at 6 weeks of age for 6 weeks. The treatment timeline is shown. **(B)** Representative images of spleen and cLNs from each treatment group and quantification of spleen weights is shown (n = 6 BL/10 sham, 6 BL/10 Imq-treated, 8 NOD.B10 sham, and 9 NOD.B10 Imq-treated mice). **(C)** Spleens and cLNs of sham (n = 6 BL/10 and 9 NOD.B10) or Imq-treated mice (n = 6 BL/10 and 7 NOD.B10) were harvested and flow cytometry was performed. Cells were gated on B220 and expression of TLR7 is shown. The percentages of B220^+^, TLR7^+^ B cells are quantified for spleen and cLNs, respectively. **(D)** Cells were gated on B220, and the percentages of cells in spleen and cLNs expressing TLR7 and CD69 are shown. Horizontal lines represent the mean and SEM (NS, non-significant, *p < 0.05, ***p < 0.001, and ***p < 0.0001).

To determine if TLR7 agonism accelerates pSS pathogenesis, we administered the TLR7 agonist Imq or sham base cream to pre-disease NOD.B10 females. Parallel experiments were performed in age and sex-matched BL/10 mice. An overview of the treatment timeline is provided in **Figure 1A**. BL/10 mice were treated in 3 individual groups, and NOD.B10 mice were treated in 4 groups. Data from each strain and treatment group were pooled from multiple experiments. An overview of the treatment groups is provided in **Supplementary Table 1**. Imq-treated BL/10 and NOD.B10 strains exhibited splenomegaly and cervical lymphadenopathy compared to sham-treated controls, and NOD.B10 mice had significantly higher spleen weights than sham-treated counterparts (p = 0.0001). BL/10 mice, in contrast, showed no difference in spleen weights between sham and Imq-treated groups (**Figure 1B**).

Since B cells are integral to pSS pathogenesis and TLR7 is highly expressed in the B cell compartment, we sought to determine if B cell TLR7 expression was altered following Imq treatment. There were no significant differences in the percentage of splenic B cells expressing TLR7 between the sham or Imq-treated strains in BL/10 or NOD.B10 mice. The percentage of B cells expressing TLR7 was upregulated in Imq-treated NOD.B10 mice, however, as compared to B cells derived from Imq-treated BL/10 controls (p = 0.01) (**Figure 1C**). The percentage of TLR7^+^ cLN B cells was similar between sham and Imq-treated BL/10 mice, although Imq treatment caused an increase in this population in pSS mice as compared to sham-treated NOD.B10 controls (p = 0.03). Additionally, the cLN B cell population derived from Imq-treated NOD.B10 mice that expressed TLR7 was expanded compared to B cells derived from Imq-treated BL/10 mice (p = 0.009) (**Figure 1C**). Finally, the percentage of activated splenic and cLN B cells expressing TLR7 did not differ between sham and Imq-treated groups in both BL/10 or NOD.B10 mice (**Figure 1D**), although the percentage of activated TLR7^+^ splenic B cells derived from sham-treated NOD.B10 mice was elevated as compared to those derived from sham-treated BL/10 females (p = 0.02). In addition, the percentage of activated TLR7^+^ splenic B cells was elevated in Imq-treated pSS mice and compared to their BL/10 counterparts (p = 0.01) (**Figure 1D**). Finally, activated TLR7^+^ splenic and cLN B cells derived from Imq-treated NOD.B10 animals were expanded as compared to those derived from Imq-treated BL/10 mice (p = 0.0005) (**Figure 1D**). Thus, TLR7 agonism induces cervical lymphadenopathy, splenomegaly, and expansion of TLR7-expressing B cells in secondary lymphoid organs, and these changes are more pronounced in pSS mice as compared to healthy controls.

### 3.2 TLR7 agonism induces sialadenitis, dacryoadenitis and loss of salivary flow

**Figure 2:**
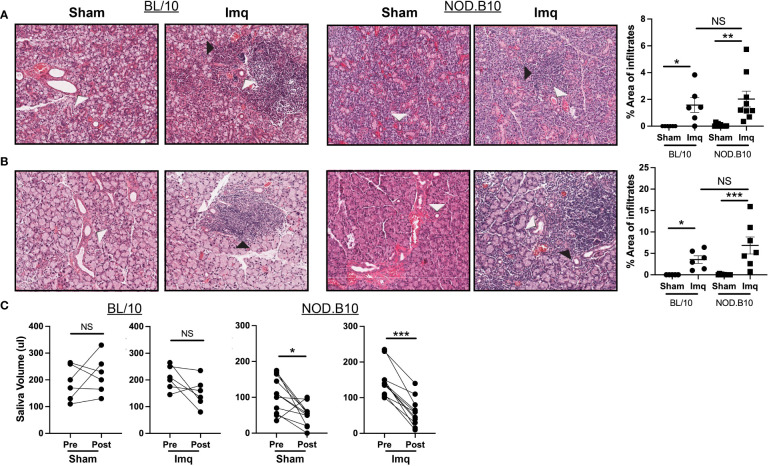
pSS mice treated with Imq exhibit robust lymphocytic infiltration of exocrine tissues and loss of salivary flow. **(A)** SMG and **(B)** lacrimal tissues were harvested from sham (n = 10) or Imq-treated female mice (n = 9) and from sham (n = 6) or Imq-treated age and sex-matched controls (n = 6). One representative photomicrograph is shown from each group. White arrows indicate exocrine gland ducts and black arrows indicate inflammation. Lymphocytic infiltration was quantified using ImageJ. Horizontal lines represent the mean and SEM **(C)** Stimulated saliva was collected prior to treatment and at the conclusion of the experiment from BL/10 and NOD.B10 mice. (NS = non-significant, *p < 0.05, **p < 0.001, ***p < 0.001).

We next evaluated exocrine gland inflammation and loss of salivary flow in each of the treatment groups, as these are hallmarks of pSS. Sialadenitis in the submandibular gland was increased in BL/10 and NOD.B10 mice that received Imq treatment as compared to sham-treated controls, (p = 0.01 and p = 0.003 respectively). Of note, the parotid glands were spared from inflammation in all treatment groups, as lymphocytic infiltration was only observed in 1 NOD.B10 Imq-treated mouse (**Supplementary Figure 1**). Dacryoadenitis was enhanced in BL/10 and NOD.B10 mice that received Imq treatment as compared to sham-treated controls (p = 0.02 and p = 0.001, respectively), although there were no differences observed in either salivary or lacrimal inflammation between BL/10 and NOD.B10 mice that received Imq (**Figures 2A, B**).

To examine whether salivary gland function was altered following TLR7 activation, we quantified salivary production in all groups at pre- and post-treatment time points (**Figure 1A**). Salivary flow remained unchanged in BL/10 animals that received either sham cream or Imq (**Figure 2C**). In pSS mice, both sham and Imq-treated animals lost salivary flow over time (p = 0.01 and p < 0.0001, respectively). These changes, however, were more pronounced in animals that received Imq as compared to the sham treatment group (**Figure 2C**). Therefore, TLR7-mediated signals mediated exocrine inflammation and salivary hypofunction.

### 3.3 TLR7 agonism induced pulmonary and renal inflammation in pre-disease NOD.B10 mice

**Figure 3:**
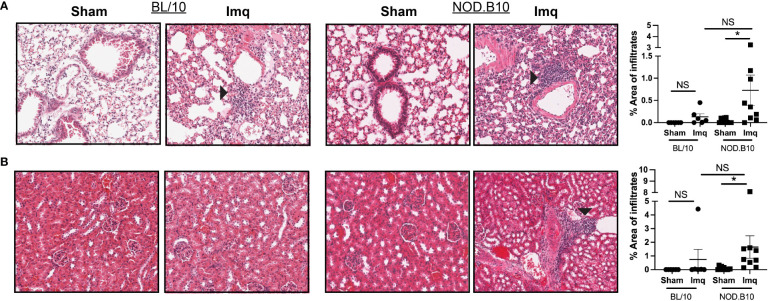
TLR7 agonism induces significant pulmonary and renal inflammation in pSS mice. **(A)** Lung and **(B)** kidney tissues were harvested from sham (n = 10) or Imq-treated female pSS mice (n = 9) and from sham (n = 6) or Imq-treated age and sex-matched controls (n = 6). One representative photomicrograph is shown from each group. Lymphocytic infiltration was quantified using ImageJ and black arrows indicate tissue inflammation. Horizontal lines represent the mean and SEM (NS = non-significant, *p < 0.05).

To determine if TLR7 stimulation induces systemic inflammation in pSS mice that is characteristic of pSS patients, we assessed pulmonary and renal inflammation in each of the treatment groups. There were no significant differences in the area of lymphocytic infiltration in either lung or kidney tissue in BL/10 mice treated with Imq as compared to strain-matched sham controls (**Figures 3A, B**). In contrast, TLR7 activation induced interstitial pneumonitis and nephritis in pSS mice (p = 0.04 and 0.02, respectively), although there was no difference in inflammation in either tissue between Imq-treated BL/10 and NOD.B10 mice (**Figures 3A, B**). All of the Imq-treated NOD.B10 mice displayed perivascular lymphoplasmacytic inflammation in the kidney, concentrated at the pelvis but tracking into the medulla and cortex in some cases (**Figure 3B**). In addition, kidneys from 3 of the NOD.B10 Imq-treated mice had evidence of glomerular damage (proteinuria), which was scored as minimal (n = 1/9), mild (n = 1/9), or severe (n = 1/9). None of the Imq-treated BL/10 mice exhibited glomerular damage. Therefore, TLR7 agonism drives systemic inflammation that is characteristic of pSS.

### 3.4 Total and ANA-specific antibodies are increased in pSS mice following TLR7 activation

**Figure 4:**
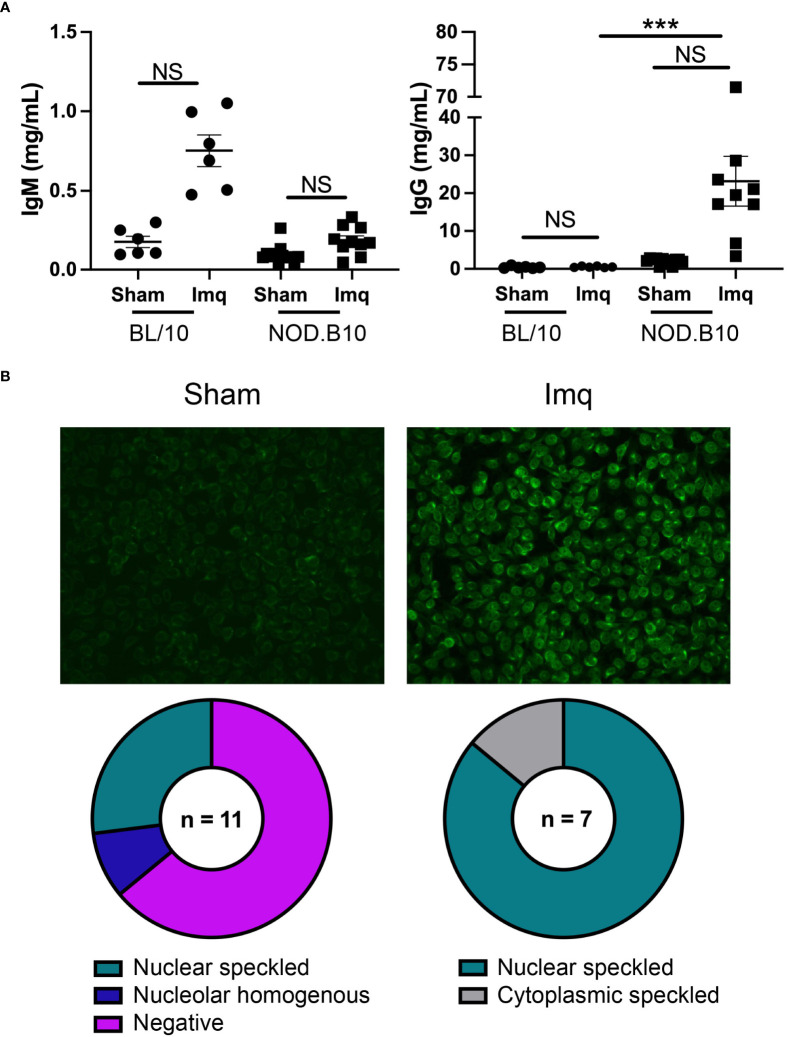
NOD.B10 mice treated with Imq demonstrate high IgG titers and ANA levels. Sera were harvested from NOD.B10 sham (n = 10) and Imq-treated female mice (n = 9) and from BL/10 sham (n = 6) and Imq-treated female BL/10 mice (n = 6) by cardiac puncture following euthanasia. **(A)** Total IgM and IgG titers were quantified by ELISA. Horizontal lines represent the mean and SEM (NS, non-significant, ****p < 0.0001). **(B)** Anti-nuclear autoantibodies were detected by HEp2 staining. One representative image is shown from each group.

We next assessed total and ANA-specific antibodies in sham and Imq-treated BL/10 and NOD.B10 mice. There were no differences in total IgM or IgG titers between sham and Imq-treated strains in both BL/10 and NOD.B10 mice. We noted a significant increase, however, in total IgG titers when comparing Imq-treated NOD.B10 mice to their BL/10 counterparts (p = 0.0003) (**Figure 4A**). We next performed HEp2 assays to evaluate ANA-specific IgG antibodies in the sera of pSS mice. Of note, we did not carry out these analyses in the BL/10 treatment groups, as IgG titers were low and remained unchanged following Imq treatment. We observed negligible ANAs in most of the sham-treated pSS mice (7/11), although a minority of animals displayed nucleolar homogenous (1/11) or nuclear speckled patterns (3/11) (**Figure 4B**). We then analyzed the Imq-treated pSS mice. In contrast, all animals displayed ANA-specific IgG autoantibodies. The majority of the sera examined displayed a nuclear speckled pattern (6/7), while a cytoplasmic speckled pattern was observed in one of mice examined (1/7) (**Figure 4B**). Thus, TLR7 agonism induced ANA production in Imq-treated pSS mice, and the patterns observed were consistent with RNA-associated autoantigens.

### 3.5 RNA-associated autoantigens are increased in pSS mice following Imq treatment

**Figure 5:**
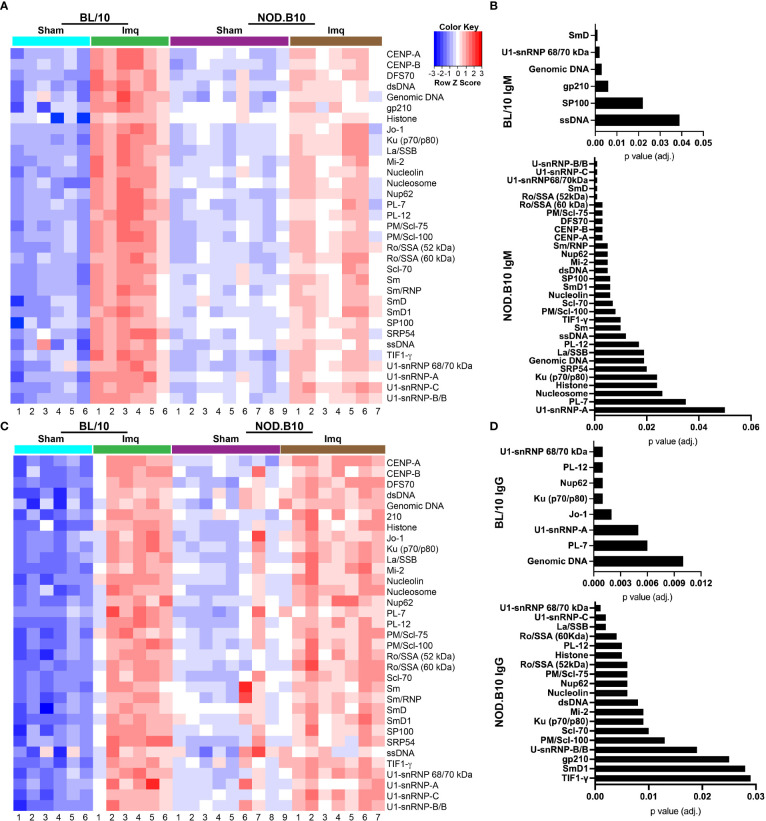
Autoantigen arrays reveal enrichment of autoantibodies in the sera of Imq-treated NOD.B10 mice. Sera were harvested from NOD.B10 sham (n = 9) or Imq-treated female mice (n = 7) by cardiac puncture following euthanasia. Sera were harvested similarly from BL/10 sham (n = 6) or Imq-treated female BL/10 mice (n = 6). Autoantigen arrays were performed and heatmaps for ANA-specific **(A)** IgM and **(C)** IgG are shown. ANA-specific **(B)** IgM and **(D)** IgG that was significantly enriched in Imq-treated BL/10 and NOD.B10 mice as compared to sham-treated strain-matched controls is shown.

To confirm and extend the ELISA and HEp2 studies, we performed autoantigen arrays on sera derived from sham or Imq-treated BL/10 and NOD.B10 mice. We focused our analyses on ANA-specific IgM and IgG (**Figure 5**). We first examined whether ANAs were elevated in sera from BL/10 mice treated with Imq as compared to sham controls. We found IgM autoantibodies directed against SmD (p = 0.001), ssDNA (p = 0.04), SP100 (p = 0.02), gp210 (p = 0.006), genomic DNA (p = 0.003), and U1-snRNP 68/70 kDa (p = 0.002) were elevated in Imq-treated mice (**Figures 5A, B**). Additionally, IgG autoantibodies directed against genomic DNA (p = 0.01), PL-7 (p = 0.006), U1-snRNP-A (p = 0.005), Jo-1 (p = 0.002), Ku (p70/p80) (p = 0.001), Nup62 (p = 0.001), PL-12 (p = 0.001), and U1-snRNP 68/70 kDa (p = 0.001) were elevated in Imq-treated BL/10 mice (**Figures 5C, D**). We next examined autoantibodies in sham and Imq-treated NOD.B10 mice. As expected, we found enrichment of numerous IgM and IgG specific ANAs in the sera from NOD.B10 mice, and many of them recognized ribonuclear proteins. Indeed, IgM directed against U1-snRNP-A (p = 0.05), PL-7 (p = 0.04), nucleosome (p = 0.03), histone (p = 0.02), Ku (p70/p80) (p = 0.02), SRP54 (p = 0.02), genomic DNA (p = 0.02), La/SSB (p = 0.02), PL-12 (p = 0.02), ssDNA (p = 0.01), TIF1-γ (p = 0.01), Sm (p = 0.01), PM/Scl-100 (p = 0.008), Scl-70 (p = 0.007), SP100 (p = 0.006), nucleolin (p = 0.006), SmD1 (p = 0.006), Nup62 (p = 0.005), dsDNA (p = 0.005), Mi-2 (p = 0.005), Sm/RNP (p = 0.005), CENP-A (p = 0.003), CENP-B (p = 0.003), PM/Scl-75 (p = 0.003), Ro/SSA (60 kDa) (p = 0.003), DFS70 (p = 0.003), U1-snRNP 68/70 kDa (p = 0.001), Ro/SSA (52 kDa), U1-snRNP-C (p = 0.001), SmD (p = 0.001), and U1-snRNP-B/B were enriched in Imq-treated mice as compared to sham controls (**Figures 5A, B**). Finally, ANA-specific IgG was also elevated in the NOD.B10-treated mice, as TIF1-γ (p = 0.03), SmD1 (p = 0.03), gp210 (p = 0.03), U-snRNP-B/B (p = 0.02), PM/Scl-100 (p = 0.01), Scl-70 (p = 0.01), Ku (p70/p80) (p = 0.009), Mi-2 (p = 0.009), dsDNA (p = 0.008), nucleolin (p = 0.006), Nup62 (p = 0.006), PM/Scl-75, (p = 0.006), Ro/SSA (52 kDa) (p = 0.006), Histone (p = 0.005), PL-12 (p = 0.005), Ro/SSA (60 kDa) (p = 0.004), La/SSB (p = 0.002), U1-snRNP-C (p = 0.002), and U1-snRNP 68/70 kDa (p = 0.001) were elevated. Of note, there were no differences observed between Imq-treated BL/10 and NOD.B10 animals in either IgM or IgG autoreactivity, although both ANA-specific IgM and IgG were elevated in NOD.B10 sham-treated animals as compared to analogous BL/10 mice (**Supplementary Figure 2**).

### 3.6 TLR7 agonism resulted in the expansion of activated/memory T cells and T-bet^+^ B cells

Since autoantibodies were elevated in pSS mice following Imq treatment, we performed studies to evaluate CD4^+^ and CD8^+^ T cell populations and T-bet^+^ B cells in the spleens and cLNs of each treatment group. There were no differences in the percentage of CD4^+^ T cells in either the spleens or cLNs from derived from BL/10 or NOD.B10 mice in any of the groups (**Supplementary Figures 3A, C**). In addition, the percentage of splenic CD8+ T cells was unchanged between BL/10 sham and Imq-treated animals, although this population was significantly diminished in the Imq-treated NOD.B10 mice as compared to sham-treated controls (p = 0.0004) (**Supplementary Figure 3B**). In the cLNs, the percentage of CD8^+^ T cells was decreased significantly in both Imq-treated NOD.B10 and BL/10 mice as compared to sham-treated controls of the same strain (p = 0.04 and p = 0.01, respectively) (**Supplementary Figure 3D**).

**Figure 6:**
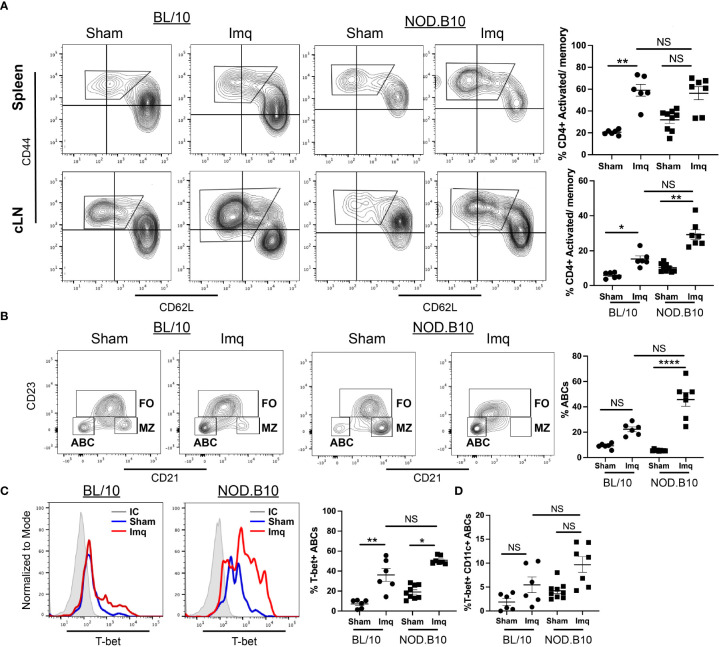
Activated/memory T cells and T-bet-expressing B cells are expanded in secondary lymphoid organs of pSS mice. Spleens and cLNs of sham (n = 9) or Imq-treated NOD.B10 mice (n = 7) and from sham (n = 6) or Imq-treated age and sex-matched controls (n = 6) were harvested and flow cytometry was performed. **(A)** Cells were gated on CD4 and CD44 and CD62L expression is shown. The percentages of activated/memory T cells (CD4^+^, CD44^+^, CD62L^-^) from spleen and cLNs are shown. **(B)** The percentages of ABCs (B220^+^, CD21^-^, CD23^-^) were quantified. ABCs were gated and expression of **(C)** T-bet and **(D)** Tbet/CD11c are shown. Plots from one representative animal from each group is shown. Horizontal lines represent mean and SEM (NS, non-significant, *p < 0.05, **p < 0.01, ****p < 0.0001).

We next assessed CD4^+^ and CD8^+^ activated/memory B cells in spleens and cLNs. We found that the percentage of splenic activated/memory CD4^+^ T cells was increased significantly in Imq-treated BL/10 mice compared to the sham controls (p = 0.003), although there was no difference in this population in spleens derived from sham and Imq-treated NOD.B10 mice. Moreover, the percentage of splenic activated/memory CD4^+^ T cells was similar in Imq-treated BL/10 and NOD.B10 animals (**Figure 6A**). In contrast, cLN activated/memory CD4^+^ T cells were expanded in both BL/10 and NOD.B10 Imq-treated mice as compared to their sham-treated counterparts (p = 0.02 and p = 0.01, respectively) (**Figure 6A**). Of note, there was no significant difference the activated/memory CD4^+^ T cell populations in Imq-treated cLNs derived from NOD.B10 mice as compared to those derived from Imq-treated BL/10 animals (**Figure 6A**).

CD8^+^ activated/memory T cells were expanded in the spleen of BL/10 mice following Imq treatment (p = 0.0006), and no changes were observed in this population in Imq-treated NOD.B10 mice as compared to sham-treated controls (**Supplementary Figure 3E**). Finally, the percentage of activated/memory CD8^+^ T cells remained unchanged in cLNs from both BL/10 and NOD.B10 Imq-treated mice as compared to sham controls (**Supplementary Figure 3F**). In contrast, in NOD.B10 sham-treated mice showed a higher percentage of activated/memory CD8+ T cells as compared to sham-treated BL/10 mice (p = 0.004) (**Supplementary Figure 3F**).

To assess if B cell populations were altered following TLR7 agonism, we assessed the percentage of total B cells, as well as the Fo and MZ B cell populations in our treatment groups. We found that the percentages of total splenic and cLN B cells and Fo B cells in the spleen did not differ between sham and Imq-treated strains in both BL/10 and NOD.B10 mice (**Supplementary Figures 4A, B, D**). Additionally, there was no change in the percentage of MZ B cells between sham and Imq-treated BL/10 mice. In contrast, Imq-treated MZ B cells from NOD.B10 mice were decreased dramatically as compared to those from NOD.B10 sham controls (p < 0.0001) (**Supplementary Figure 4C**). Additional studies were performed to assess BAFF levels in the sera of sham and Imq-treated mice. We found that Imq-treated NOD.B10 mice had elevated serum BAFF levels as compared to their sham-treated counterparts (p = 0.049). While BAFF levels tended to be higher in the BL/10 Imq-treated mice as compared to sham-treated controls, this difference did not reach statistical significance (p = 0.053) (**Supplementary Figure 4E**). Finally, serum β2-microglobulin levels were assessed. We found that β2-microglobulin was elevated in the sera of Imq-treated NOD.B10 mice as compared to sham-treated NOD.B10 controls (p = 0.02). There was no difference between sham and Imq-treated BL/10 mice, or between Imq-treated BL/10 and NOD.B10 mice (**Supplementary Figure 4F**).

We next assessed the splenic ABC populations in sham and Imq-treated mice. The percentage of ABCs (B220^+^, CD21^-^, CD23^-^) in sham and Imq-treated BL/10 animals was similar (**Figure 6B**). In contrast, we observed a significant expansion of ABCs derived from Imq-treated NOD.B10 mice as compared to sham controls (p < 0.0001) (**Figure 6B**). We then assessed the percentage of ABCs that expressed T-bet and CD11c, as these markers are characteristic of ABCs, and identify distinct subsets within this population. We found that T-bet expression was significantly higher in ABCs derived from Imq-treated strains than sham controls in both BL/10 and NOD.B10 animals (p = 0.008 and 0.02, respectively) (**Figure 6C**). However, there was no difference in T-bet expression in ABCs derived from Imq-treated NOD.B10 mice as compared to those from Imq-treated BL/10 mice (**Figure 6C**). Lastly, there were no significant differences in the percentage of ABCs that expressed T-bet and CD11c between Imq-treated strains and sham controls in both BL/10 and NOD.B10 animals (**Figure 6D**). Finally, we examined the percentage of splenic monocytic cells (B220^-^, CD11c^+^) in each of the treatment groups. We found no differences between the percentage of CD11c^+^ cells in spleens derived from BL/10 mice when we compared sham and Imq-treated mice. In addition, the percentage of CD11c^+^ cells was similar between Imq-treated BL/10 and NOD.B10 mice. When we examined the CD11c-expressing monocytes in NOD.B10 mice, however, we found that Imq-treated mice exhibited higher levels of this population as compared to sham-treated counterparts (p = 0.007) (**Supplementary Figure 5**). Thus, treatment of pSS mice with a TLR7 agonist results in the expansion of activated/memory CD4^+^ T cells, T-bet^+^ B cells, and CD11c^+^ monocytes.

## 4 Discussion

### 4.1 TLR7 signaling mediates local and systemic pSS disease

Our study revealed that pre-disease stage pSS mice develop accelerated local and systemic pSS manifestations in response to treatment with the TLR7 agonist Imq. Salivary and lacrimal inflammation were increased as compared to sham-treated NOD.B10 controls in response to Imq treatment, although the area occupied by lymphocytes in exocrine tissue did not differ between Imq-treated BL/10 and NOD.B10 mice (**Figures 2A and B**). Salivary flow, however, was reduced in pSS mice treated with Imq, while saliva production remained unchanged in healthy controls following Imq administration (**Figure 2C**). In agreement with the literature, these data suggest that salivary hypofunction is not mediated exclusively by the presence of lymphocytic infiltrates within salivary tissue, and other factors, such as apoptosis and anti-muscarinic 3 receptor autoantibodies, likely contribute to the dryness observed (48–51). This finding also raises the intriguing possibility that TLR7 activation in the parenchymal compartment could be a prime driver of the salivary dysfunction observed in pSS mice, although further studies in TLR7-deficient models are needed to establish this conclusively.

In contrast to our findings in exocrine tissue, inflammation was increased in the lungs and kidneys from pSS mice in response to Imq treatment, and this was not observed in the analogous BL/10 treatment group (**Figure 3**). Whether this observation relates to intrinsic differences in the pulmonary and renal microenvironments in NOD.B10 mice, and/or whether this inflammation is driven primarily by TLR7-mediated peripheral immune cell activation remains to be determined.

The exocrine-specific and extraglandular disease manifestations were similar when we compared the Imq-treated NOD.B10 mice to NOD.B10 female mice at the clinical disease stage that develop pSS spontaneously. Differences, however, were observed in the antibody repertoire of Imq-treated mice as compared to pSS mice as the clinical disease stage. Indeed, HEp2 staining of NOD.B10 Imq-treated mice revealed that their sera displayed primarily a speckled pattern, while only a minority of the serum samples derived from NOD.B10 mice at 6 months of age exhibited a speckled pattern (4/15 animals) (7). In addition, IgM titers were not increased in Imq-treated animals, while these are elevated in NOD.B10 mice that developed disease spontaneously (7, 39). Finally, cervical lymphadenopathy and splenomegaly were observed in Imq-treated mice, while this is not characteristic of NOD.B10 females that develop the disease spontaneously (7). While we do not know the reason for these differences, we suspect that in spontaneous disease many other factors besides TLR7 activation contribute to disease development, as additional MyD88-dependent and -independent signaling cascades are implicated (6, 52–55). Thus, while TLR7 agonism recapitulates many features of pSS disease that develop spontaneously in this model, other inflammatory networks likely contribute to disease. **Table 1** provides a comparison of the findings in Imq-treated NOD.B10 mice with those observed in NOD.B10 mice at the clinical disease stage.

**Table 1:** Comparison of Disease Manifestations between Imq-treated NOD.B10 mice and clinical disease stage NOD.B10 females.

	Imq-induced	Clinical disease (spontaneous)
Sialadenitis - SMG	Yes	Yes
Sialadenitis- Parotid	Negligible	Negligible
Hyposalivation	Yes	Yes
Dacryoadenitis	Yes	Yes
Nephritis	Yes	Yes
Pneumonitis	Yes	Yes
Elevated serum IgM	No	Yes
Elevated serum IgG	Yes	Yes
Autoantibodies	Yes	Yes
Splenomegaly	Yes	No
Cervical Lymphadenopathy	Yes	Minimal

### 4.2 TLR7 signaling cascades are critical for the development and expansion of ABCs

Activation of distinct B cell subsets by TLR7 ligands is integral to autoimmunity, and elegant studies conducted over the past decade have revealed an important role for ABCs in mouse models of lupus pathogenesis (17, 56–59). Of direct relevance to the current study, TLR7 is required for ABC expansion in the context of lupus (57, 58), and B cells that express T-bet drive lupus-like disease in mice (56). Importantly, following stimulation with a TLR7 agonist, ABCs derived from healthy mice secrete heightened IgM and IgG (58). Additionally, ABCs from NZB/WF1 lupus mice produce high levels of IgG autoantibodies as compared to other B cell subsets derived from the same strain (58), and corroborative *in vivo* studies demonstrate that depletion of the ABC subset leads to a reduction in autoantibody titers (58). Subsequent work revealed that T-bet^+^ B cells mediate autoantibody production in the context of lupus (56). Significantly, ablation of T-bet^+^ B cells diminished germinal centers, protected against kidney damage, and reduced B and T cell activation (56), demonstrating that ABCs play an essential role in autoimmunity.

### 4.3 T-bet^+^ B cells are expanded in pSS mice in response to TLR7 activation

We found high titers of total and autoreactive IgG in pSS mice following TLR7 agonism (**Figures 4**, **5**). This was accompanied by dramatic expansion of splenic ABCs (B220^+^, CD23^-^, CD21^-^, T-bet^+^ B cells) that was greater than that observed in sham controls and BL/10 Imq-treated animals (**Figures 4A**, **6B, C**). Moreover, stimulation of pre-disease pSS females with Imq resulted in elevated percentages of B cells expressing TLR7 in cLNs (**Figure 1C**). Furthermore, the percentage of activated B cells that expressed TLR7 was expanded in the spleens and cLNs of Imq-treated pSS mice as compared with the BL/10 treatment group that also received Imq (**Figure 1D**). Activated/memory CD4^+^ T cells were also increased in the cLNs of Imq-treated mice (**Figure 6A**). It is interesting to speculate that this T cell expansion may be driven by the elevated percentages of T-bet^+^ B cells observed in pSS mice following treatment.

Evidence for ABC-driven T cell expansion in autoimmunity is provided by data from a lupus model that exhibits excessive ABC accumulation (60). In this model, the ABC subset induced expansion and heightened proliferation of CD44^+^CD62L^-^ T effector memory cells as compared to the Fo B cell subset, and this was mediated by the potent antigen presentation function exhibited by ABCs (60). Altogether, this work suggests that TLR7 activation in the B cell compartment is integral to pSS disease progression, although further studies are needed to determine whether TLR7-induced T-bet^+^ B cells are the prime driver of disease in our model, or whether organ-specific TLR7 activation of stromal and parenchymal cells also mediates pathology. Of note, these possibilities are not mutually exclusive, as TLR7 signals in diverse tissue microenvironments may activate distinct signaling networks that govern specific disease manifestations.

Our work is relevant to the human disease, as B cells that share both phenotypic and functional properties with murine ABCs are dysregulated in human autoimmunity (59). Of particular significance, CD19^+^, CD21^-/lo^ B cells are expanded in patients with pSS (61, 62). These cells are reminiscent of ABCs characterized in mice, as they are enriched in reactivity to nuclear and cytoplasmic autoantigens and they are responsive to TLR agonism (61, 62). In addition, CD21^lo^ B cells derived from patients with autoimmunity can serve as antigen presenting cells, as these cells express high levels of CD80, CD86 and HLA-DR (63–65). Recent work demonstrated that CD21^lo^ B cells consistently express high levels of T-bet and differentiate in response to TLR agonism or T cell help (66). While the relevance of TLR signaling in the development and subsequent activation of the CD21^lo^ B cell subset requires further study, these data suggest that autoreactive CD21^lo^ B cells could be an important mediator of T cell activation in human autoimmunity, including pSS (66).

### 4.4 ABCs exhibit TLR7-driven sexual dimorphism in lupus and this likely contributes to the female disease predilection in pSS

It is important to note that pSS is primarily seen in middle-aged females, and the diagnosis in males is considerably more rare (1). Although the underlying molecular mechanisms that govern this striking sex predilection are not well-understood at present, emerging data suggest that improper X-chromosome inactivation (XCI) and subsequent gene dosage affects may account for the high disease prevalence observed in women (67). Accordingly, TLR7 is expressed on the X-chromosome and XCI escape in lymphocytes from SLE patients is well documented, resulting in elevated TLR7 expression (67). Recent work in mice that develop a lupus-like disease found that ABCs were preferentially expanded in females (68). In addition, female-derived ABCs from this model showed an enhanced IFN-gene signature as compared to ABCs derived from male counterparts. Intriguingly, overexpression of TLR7 in males reversed the sex-bias observed and resulted in heightened ABC-mediated pathology, culminating in lung pathology and diminished overall survival (68). While no studies to date have examined whether TLR7 expression and function are altered between males and females in the context of pSS, additional work is needed to determine whether ABCs are preferentially expanded in females and whether this may be in response to TLR-medicated signaling, as this may represent a previously unappreciated disease mechanism that underlies the high prevalence of disease observed in women.

### 4.5 Ro and La may serve as endogenous TLR7 ligands in pSS

Currently, the etiology of pSS is poorly understood and early disease events are not clearly defined. Therefore, it is of critical importance to understand how TLR7 becomes activated in the context of pSS. MRL/lpr lupus mice that lack TLR7 exhibit disease amelioration and diminished antibodies against RNA-associated antigens (69). Corroborative studies in a lupus mouse model revealed that increasing TLR7 gene dosage augments production of autoantibodies directed against RNA (70). These findings are relevant to human disease, as most patients with pSS and SLE display autoantibodies to Ro (SSA) and La (SSB) (71, 72). Both Ro and La are ribonuclear proteins that form complexes with RNA (73). It is hypothesized that the RNA in these complexes serves as an endogenous adjuvant to activate TLR7-expressing B cells and drive plasma cell differentiation. This may explain, at least in part, the extremely high titers of these autoantibodies observed in SLE patients (74, 75). It is interesting to note that NOD.B10 mice treated with Imq demonstrated enrichment of IgG autoantibodies directed against RNA-associated antibodies, such as Ro, La, U1-snRNP 68/70 kDa, U1-snRNP-C, U-snRNP-B/B, and SmD1 (**Figure 5**). Thus, it is possible that TLR7 activation of B cells may occur through Ro and La-mediated activation of B cells in the context of human disease, although further studies are needed to identify clinically-relevant sources of TLR7 activation in pSS patients.

## 5 Conclusion

In conclusion, TLR7 agonism accelerates both local and systemic disease manifestations in a mouse model of pSS. This work has important therapeutic relevance, as targeting of TLR7-dependent signaling networks may be efficacious in reducing B cell activation and tissue-specific inflammation that is characteristic of disease.

## Data availability statement

The datasets presented in this study can be found in online repositories. The names of the repository/repositories and accession number(s) can be found on: https://www.ncbi.nlm.nih.gov/geo/, GSE212467.

## Ethics statement

The animal study was reviewed and approved by University of Buffalo Institutional Animal Care and Use Committee.

## Author contributions

JiK conceived of the work, wrote the manuscript and performed experiments. AP, EK, JeK, and JiK critically edited the manuscript and performed experiments. CZ performed the autoantigen arrays and normalized the data. JM critically edited the manuscript and analyzed the autoantigen arrays. All authors contributed to the article and approved the submitted version.

## Supplementary material

Supplementary Figure 1 Parotid gland tissue from both BL/10 and NOD.B10 mice exhibits negligible inflammation following Imq administration. Parotid tissues were harvested from sham (n = 10) or Imq-treated female mice (n = 9) and from sham (n = 6) or Imq-treated age and sex-matched controls (n = 6). One representative photomicrograph is shown from each group. Lymphocytic infiltration was quantified using ImageJ. White arrows represent salivary gland ducts. Horizontal lines represent the mean and SEM (NS = non-significant).

Supplementary Figure 2 Autoantigen arrays reveal enrichment of specific autoantibodies in sham-treated NOD.B10 mice as compared to BL/10 sham-treated controls. Sera were harvested from NOD.B10 (n = 9) or BL/10 sham controls (n = 6) by cardiac puncture following euthanasia. Autoantigen arrays were performed for **(A)** IgM and **(B)** IgG. Autoantibodies that were enriched in NOD.B10 animals are shown.

Supplementary Figure 3 The percentage of CD8^+^ T cells are decreased in spleens and cLNs of Imq-treated mice. Spleens and cLNs of sham (n = 9) or Imq-treated NOD.B10 mice (n = 7) and from sham (n = 6) or Imq-treated age and sex-matched controls (n = 6) were harvested and flow cytometry was performed. The percentage of splenic and cLN **(A and C)** CD4^+^ and **(B and D)** CD8^+^ cells are shown. The percentages of activated/memory T cells (CD8^+^, CD44^+^, CD62L^-^) from **(E)** spleen and **(F)** cLNs are shown. (NS, non-significant, *p < 0.05, **p < 0.01, ***p < 0.001).

Supplementary Figure 4 Marginal zone B cells are diminished in NOD.B10 mice following TLR7 agonism. Spleens and cLNs of sham (n = 9) or Imq-treated NOD.B10 mice (n = 7) and from sham (n = 6) or Imq-treated age and sex-matched controls (n = 6) were harvested and flow cytometry was performed. The percentage of **(A)** total splenic B cells (B220^+^), **(B)** follicular B cells (B220^+^, CD23^+^, CD21^lo/-^) and **(C)** marginal zone B cells (B220^+^, CD23^-^, CD21^+^) are shown. **(D)** The percentage of B cells in the cLNs of BL/10 and NOD.B10 sham and imq-treated mice are shown. Sera were harvested and **(E)** BAFF levels were assessed in C57BL/10 sham and Imq-treated (n = 6 each) and NOD.B10 sham and Imq-treated animals (n = 10 each) following euthanasia. **(F)** Serum β2-microglobulin levels were assessed in C57BL/10 sham and Imq-treated (n = 6 each) and NOD.B10 sham and Imq-treated mice (n = 9 each) following euthanasia (NS, non-significant, *p < 0.05, ***p < 0.001).

Supplementary Figure 5 The percentages of CD11c^+^ monocytes (B220^-^, CD11c^+^) were quantified. Histogram plots from one representative animal from each group are shown. Horizontal lines represent mean and SEM (NS, non-significant, **p < 0.01).
