# Supplementary material for: TLR7 agonism accelerates disease in a mouse model of primary Sjögren’s syndrome and drives expansion of T-bet+ B cells
Source: Front Immunol. 2022 Dec 15;13:1034336. doi: 10.3389/fimmu.2022.1034336 (PMC9799719; doi:10.3389/fimmu.2022.1034336)
Supplement: Supplementary file 1 [file DataSheet_1.docx]

Supplemental Table 1: Treatment Groups

| **BL/10** | **Sham** | **Imq** |
| --- | --- | --- |
| Group 1 | 3 | 3 |
| Group 2 | 2 | 1 |
| Group 3 | 1 | 3* |
| **NOD.B10** |  |  |
| Group 1 | 2 | 2 |
| Group 2 | 3 | 3 |
| Group 3 | 1 | 1 |
| Group 4 | 5 | 5 |

*1 animal died during study
