# Supplementary figures and images for: TLR7 agonism accelerates disease in a mouse model of primary Sjögren’s syndrome and drives expansion of T-bet+ B cells
Source: Front Immunol. 2022 Dec 15;13:1034336. doi: 10.3389/fimmu.2022.1034336 (PMC9799719; doi:10.3389/fimmu.2022.1034336)

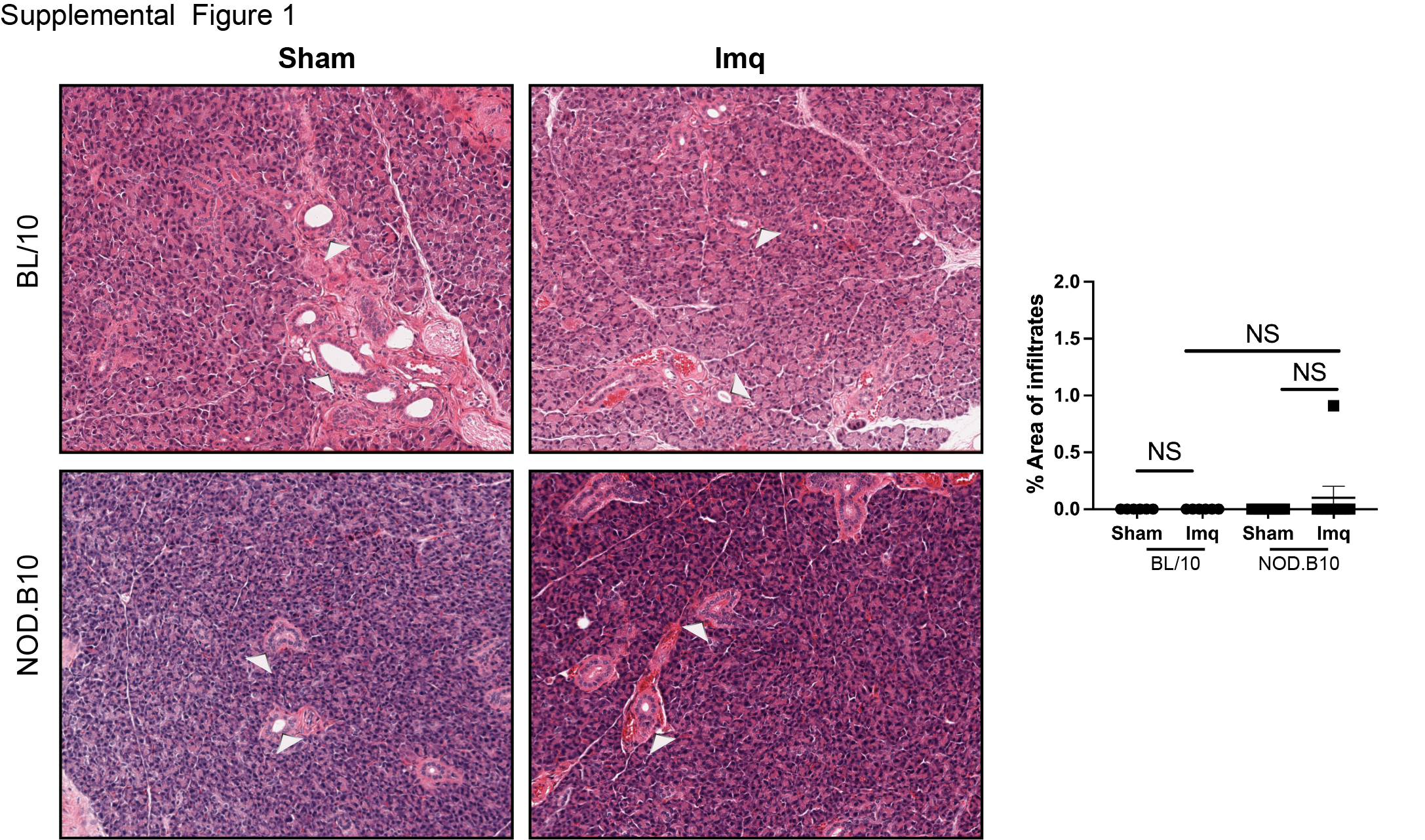

Supplement: Supplementary Figure 1 — Parotid gland tissue from both BL/10 and NOD.B10 mice exhibits negligible inflammation following Imq administration. Parotid tissues were harvested from sham (n = 10) or Imq-treated female mice (n = 9) and from sham (n = 6) or Imq-treated age and sex-matched controls (n = 6). One representative photomicrograph is shown from each group. Lymphocytic infiltration was quantified using ImageJ. White arrows represent salivary gland ducts. Horizontal lines represent the mean and SEM (NS = non-significant). [file Image_1.tif]

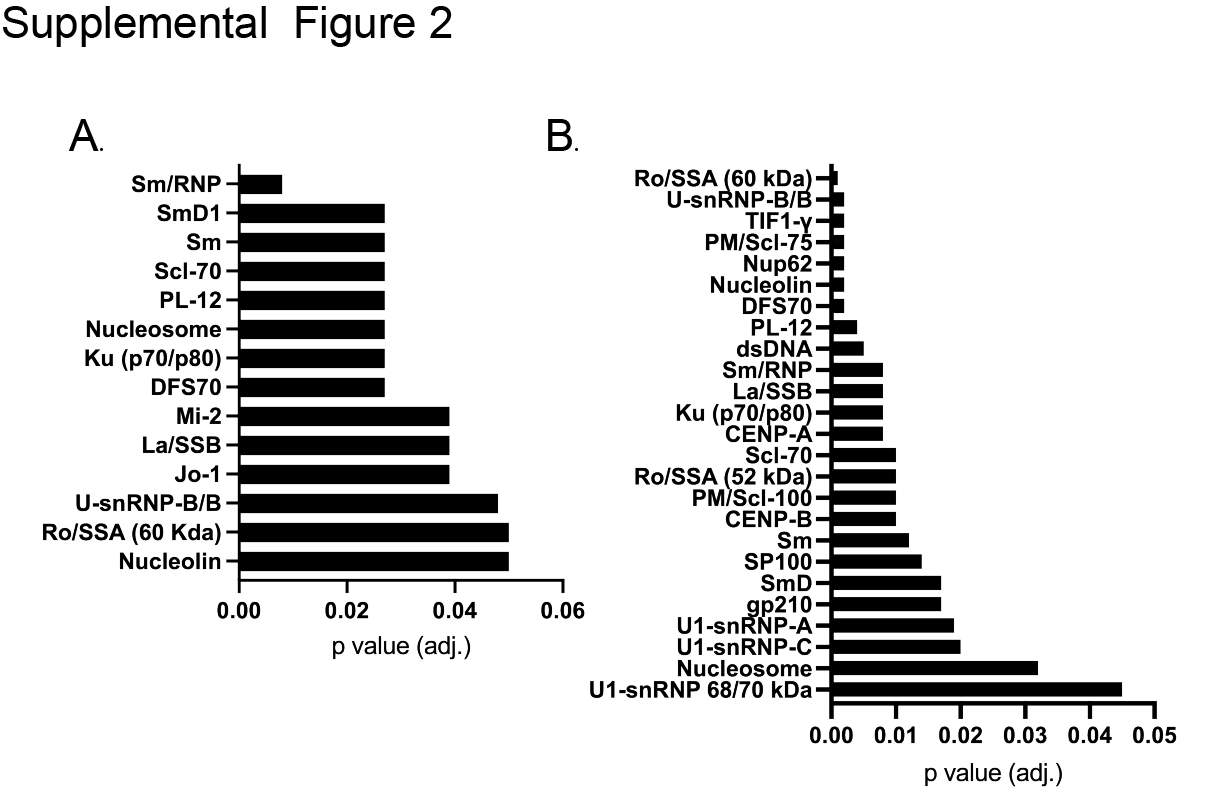

Supplement: Supplementary Figure 2 — Autoantigen arrays reveal enrichment of specific autoantibodies in sham-treated NOD.B10 mice as compared to BL/10 sham-treated controls. Sera were harvested from NOD.B10 (n = 9) or BL/10 sham controls (n = 6) by cardiac puncture following euthanasia. Autoantigen arrays were performed for (A) IgM and (B) IgG. Autoantibodies that were enriched in NOD.B10 animals are shown. [file Image_2.tif]

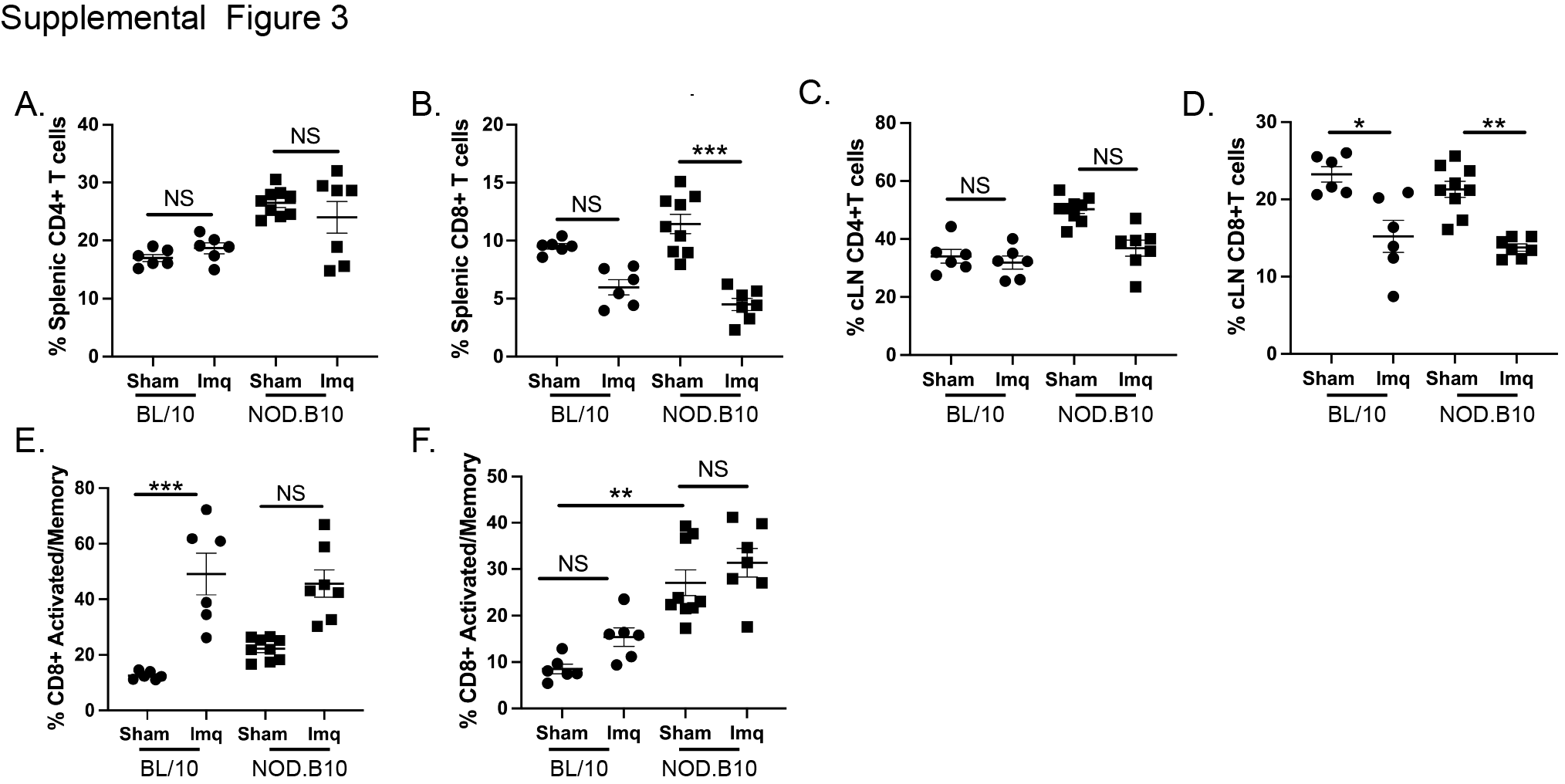

Supplement: Supplementary Figure 3 — The percentage of CD8+ T cells are decreased in spleens and cLNs of Imq-treated mice. Spleens and cLNs of sham (n = 9) or Imq-treated NOD.B10 mice (n = 7) and from sham (n = 6) or Imq-treated age and sex-matched controls (n = 6) were harvested and flow cytometry was performed. The percentage of splenic and cLN (A and C) CD4+ and (B and D) CD8+ cells are shown. The percentages of activated/memory T cells (CD8+, CD44+, CD62L-) from (E) spleen and (F) cLNs are shown. (NS, non-significant, *p < 0.05, **p < 0.01, ***p < 0.001). [file Image_3.tif]

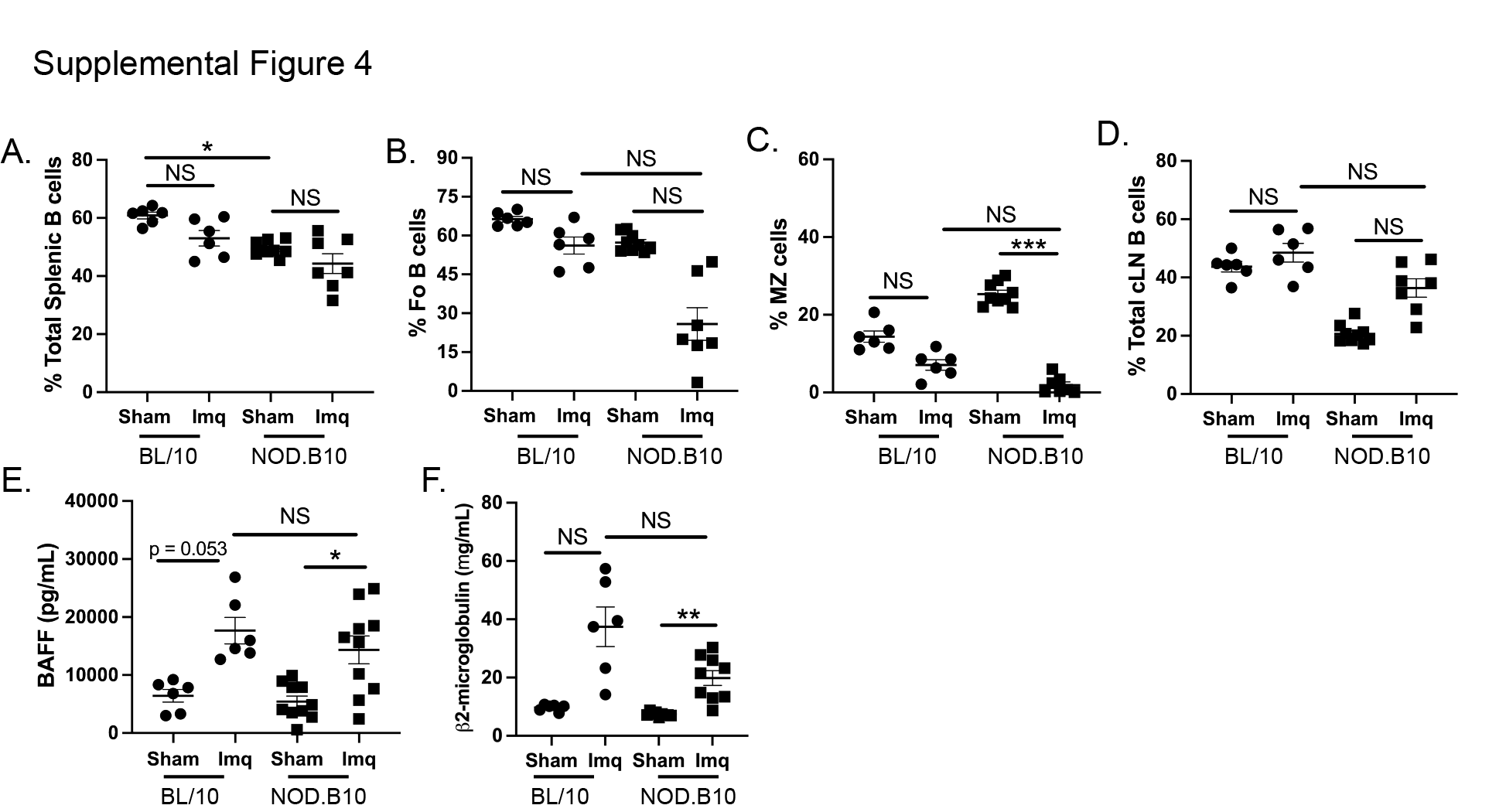

Supplement: Supplementary Figure 4 — Marginal zone B cells are diminished in NOD.B10 mice following TLR7 agonism. Spleens and cLNs of sham (n = 9) or Imq-treated NOD.B10 mice (n = 7) and from sham (n = 6) or Imq-treated age and sex-matched controls (n = 6) were harvested and flow cytometry was performed. The percentage of (A) total splenic B cells (B220+), (B) follicular B cells (B220+, CD23+, CD21lo/-) and (C) marginal zone B cells (B220+, CD23-, CD21+) are shown. (D) The percentage of B cells in the cLNs of BL/10 and NOD.B10 sham and imq-treated mice are shown. Sera were harvested and (E) BAFF levels were assessed in C57BL/10 sham and Imq-treated (n = 6 each) and NOD.B10 sham and Imq-treated animals (n = 10 each) following euthanasia. (F) Serum β2-microglobulin levels were assessed in C57BL/10 sham and Imq-treated (n = 6 each) and NOD.B10 sham and Imq-treated mice (n = 9 each) following euthanasia (NS, non-significant, *p < 0.05, ***p < 0.001). [file Image_4.tif]

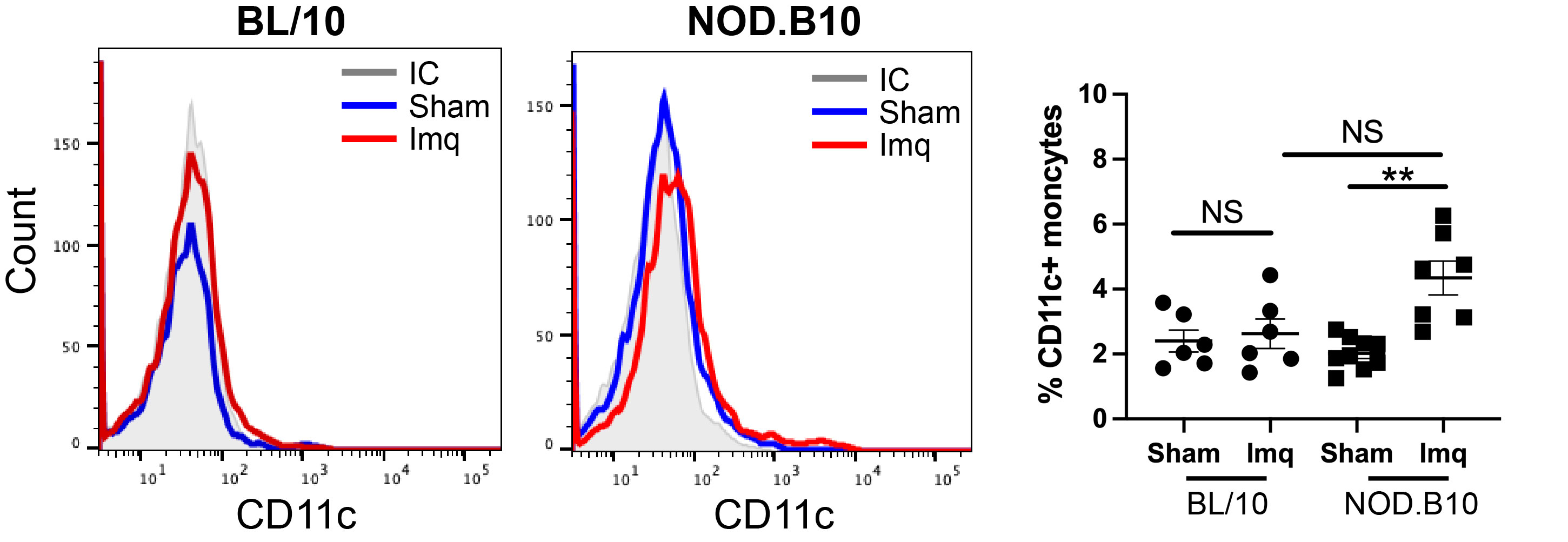

Supplement: Supplementary Figure 5 — The percentages of CD11c+ monocytes (B220-, CD11c+) were quantified. Histogram plots from one representative animal from each group are shown. Horizontal lines represent mean and SEM (NS, non-significant, **p < 0.01). [file Image_5.jpeg]
